# Supplementary material for: “The Best Home for This Paper”: A Qualitative Study of How Authors Select Where to Submit Manuscripts
Source: Perspect Med Educ. 2024 Sep 9;13(1):442–51. doi: 10.5334/pme.1517 (PMC11405847; doi:10.5334/pme.1517)
Supplement: Appendix. — Semi-structured focus group guide. [file pme-13-1-1517-s1.pdf]

Appendix: Semi-structured focus group guide

To get started, imagine that you are preparing to submit a manuscript focused on an HPE topic. Please describe how you would select your target journal.

Potential Prompts (not all questions will necessarily be asked)

- What specific factors come into play and why are they important?
  - *Likely journal impact factor (JIF) will be mentioned. If necessary, after a few minutes of discussion about the JIF state: Taking impact factor off the table, which other factors are important to you?*
  - How does your institution's promotion and tenure guidelines influence your decision?
  - In previous studies, HPE authors have said that access to their work is important. What role, if any, does a journal's open access status play?
    - What about if there is an author processing charge?
- Before submitting to a journal, what are some of the factors that you wished you knew about that are not readily accessible? *If an example is needed, you could say rejection rate.*
- What roles do your co-authors play in this decision?
  - At what point do you discuss your target journal with your team?
  - In what ways, if any, does the makeup of your team influence selection (e.g., having ECRs, someone going up for tenure/promotion, clinically based authors)
- When do you consider publishing in a specialty academic journal (e.g., Academic Pediatrics) vs. a more general HPE journal?
  - In what ways, if any, would your approach be different if you were planning to publish outside of HPE, for example in a clinical or psychology journal?
- How does your process change after a rejection? After the third rejection?
- Previous research has shown that a journal's "reputation" plays into journal selection decisions. Do you feel this is still true and if yes, how do you judge a journal's reputation?
  - What do you consider a red flag and would it stop you from submitting?
  - What are characteristics of a journal that reassure you about its integrity?
  - Where do you look for information about a journal's integrity?
  - In what ways, if any, do you guard against submitting to predatory journals?
  - How did you learn about journal integrity?
